# Hyperacute and Fulminant Guillain-Barré Syndrome Requiring Emergent Intubation

**DOI:** 10.7759/cureus.38499

**Published:** 2023-05-03

**Authors:** James Neumeister, Derrick Huang, Shane Dzluneski, Alexander M Huttleston, Christopher Megargel, Michael Falgiani, Latha Ganti

**Affiliations:** 1 Emergency Medicine, HCA Florida Ocala Hospital, Ocala, USA; 2 Emergency Medicine, Envision Physician Services, Plantation, USA; 3 Emergency Medicine, University of Central Florida College of Medicine, Orlando, USA

**Keywords:** polyneuropathy, autoimmune disease, emergent intubation, areflexia, guillain-barre syndrome (gbs)

## Abstract

Guillain-Barré syndrome (GBS) is an autoimmune-mediated acute polyneuropathy that can progress to life-threatening respiratory failure. The diagnosis and treatment of this pathology are complicated by the rarity of the disease and diversity in clinical presentation due to rarer, more dangerous subtypes of GBS. Understanding the time course of progression from onset to nadir of neurological deficits, maintaining a high index of suspicion, and close airway monitoring are essential in rapid diagnosis, securing the airway, and treatment.

## Introduction

Guillain-Barré syndrome (GBS) is an autoimmune-mediated acute polyneuropathy that is an uncommon emergency department (ED) presentation with an estimated annual incidence in the United States of 1.65 to 1.79 per 100,000 persons [1]. This syndrome encompasses a group of neuropathic pathologies characterized by progressive weakness and diminished or absent reflexes that may advance to respiratory failure in about 20-30% of cases [1-2]. GBS is believed to be secondary to an abnormal immune response involving antibodies directed against myelin sheaths or axons [1-2]. This response is typically triggered by an antecedent event up to four weeks prior to the onset of neurological symptoms in about 76% of patients [3]. These events most commonly involve upper respiratory tract infections (URI) (35%), including severe acute respiratory syndrome coronavirus 2, with a large proportion of cases also involving gastroenteritis, most notably with *Campylobacter jejuni* [1,3]. Rarer antecedent events involving surgery, immunizations, and immune checkpoint inhibitor therapy have also been described [2-4].

The most common form of GBS, acute inflammatory demyelinating polyradiculoneuropathy (AIDP), encompasses up to 90% of cases and presents with progressive, ascending motor weakness that classically begins distally in the legs and advances proximally [1-2]. There are also rarer, more rapidly progressive forms of GBS, most notably acute motor axonal neuropathy (AMAN) and acute motor and sensory axonal neuropathy (AMSAN) [4-5]. Other variants include the GQ1b syndromes, which include Miller Fisher syndrome (MFS), Bickerstaff brainstem encephalitis (BBE), and the pharyngeal-cervical-brachial variant. MFS and BBE are generally characterized by a predominant presentation with ophthalmoplegia, ataxia, and facial and bulbar motor dysfunction, as opposed to the limb weakness, due to antibodies against GQ1b gangliosides of oculomotor nerve myelin [4,6].

In the ED, the diagnosis of GBS is complicated by rarer atypical variants with hyperacute progression of neurological deficits within 48 hours [2,4,7-8]. With a hyperacute presentation, a wider differential that includes cerebrovascular accidents, acute myelopathies and polyneuropathies, neuromuscular junction disease, and tick paralysis is required. Furthermore, rapid progression of paralysis demands close monitoring for rapid airway decompensation, particularly in cases of diaphragmatic and bulbar involvement [9]. Here, we present a case of hyperacute GBS with severe dysautonomia and rapid airway decompensation requiring emergent intubation.

## Case presentation

A 20-year-old male with a history of depression and a prior suicide attempt presented to the ED with shortness of breath and generalized weakness. The patient was seen earlier that day at another ED, where he was diagnosed with URI and discharged home. He now felt worsening shortness of breath. His symptoms started the previous day when he noticed his lower legs began to feel weak, progressing to shortness of breath. He reported a brief diarrheal illness one week ago. He denied fevers, coughing, chest pain, abdominal pain, and URI symptoms. He denied drug ingestion, current suicidal ideation, recent travel, eating undercooked or canned foods, and animal bites. His immunizations were up-to-date.

On his initial vitals, the patient was afebrile. He had a heart rate of 106 bpm, respiratory rate of 20, blood pressure of 145/79 mmHg, and pulse oximetry of 99% on room air. The patient had sustained episodes of tachycardia with a heart rate in the 140s bpm that would briefly resolve and recur. The patient was anxious, slightly tachypneic, and his voice was hardly audible. He spoke in short phrases with a short inspiratory phase. His breath sounds were clear. The patient had difficulty leaning forward or sitting up due to weakness. When asked to raise his arms, he barely lifted them against gravity momentarily. His upper extremities had 3/5 muscle strength, his hips had 2/5 muscle strength, and his legs had 0/5 muscle strength. His sensation was intact and the patient had diffusely absent deep tendon reflexes

The patient was placed on supplemental oxygen and preparations were made for intubation. Neurology was consulted and intravenous immunoglobulin (IVIg) was ordered for administration at this time. Before measurement of end-tidal CO_2_, arterial blood gas, and negative inspiratory force could be performed, the patient began to experience worsening anxiety and exhaustion from increased work of breathing. Non-invasive bilevel positive airway pressure was briefly initiated and 1 mg IV lorazepam was administered; however, the patient began to have increased oral secretions. The patient was administrated etomidate and rocuronium and then intubated. A lumbar puncture was performed with a cerebrospinal fluid analysis significant for a white blood cell count of 3 cells/mm^3 ^(less than 5 is normal), glucose of 91 mg/dL (normal is 50-80 mg/dL), and protein of 86 mg/dL (normal is 15-45 mg/dL). The patient was admitted to the intensive care unit where he completed a regimen of five doses of IVIg, three sessions of plasmapheresis, and a repeated five doses of IVIg. The patient experienced a prolonged hospital stay of over one month, where he had a tracheostomy placed and was weaned off the ventilator into a trach collar. He was ultimately able to speak with a Passy Muir valve and had some improvement in extremity movement prior to being discharged to long-term rehabilitation.

## Discussion

We report a hyperacute case of GBS with rapid airway decompensation requiring emergent intubation in the ED. Our case was complicated by both a rapid progression of symptoms between 24-48 hours of the onset of neurological symptoms as well as fulminant severity at its nadir requiring intubation for airway protection [8]. Typically, about 50% of cases of GBS achieve clinical nadir by two weeks, and greater than 90% of cases achieve clinical nadir by four weeks [4,8,10]. The patient likely experienced the rarer subtype of GBS, AMAN, due to his presentation with isolated motor symptoms without sensory loss and the rapid course of illness (4-5). Representing about 5-10% of GBS cases in the United States, AMAN and AMSAN are the primary axonal variants of GBS (4-5). These variants are more frequently encountered in China, Japan, and Mexico with a predominant effect on younger patients, and a greater risk of developing prolonged paralysis and respiratory failure over a few days with a mortality rate of about 3-5% [4-5,11-12]. As reflected in our case, AMAN has a strong association with a preceding *C. jejuni* infection and antiganglioside IgG antibodies to peripheral nerve axons due to molecular mimicry, or an antibody and complement-mediated humoral immune response [11]. Compared to AIDP, there may be a more rapid progression, preservation of deep tendon reflexes (DTRs), and highly selective involvement of motor nerves [4]. AMSAN may be considered a more dangerous form of AMAN, resembling AMAN with greater axonal degeneration, sensory nerve involvement, and a worse long-term prognosis [4]. As in our case, rapidly progressive weakness (reaching nadir rapidly), bulbar and facial weakness, and autonomic dysfunction are strong indicators for the potential need for intubation to secure the airway, Immunomodulatory therapy, and disposition to ICU level of care for frequent monitoring and airway support (9).

In the ED, diagnosis of GBS is predominately clinical. GBS cases should present with progressive weakness involving the extremities and/or the trunk, bulbar, facial, or ocular muscles in addition to areflexia or decreased DTRs [13]. Diagnosis is supported by dysautonomia, mild sensory dysfunction, and absence of fever at symptom onset. Additionally, cerebrospinal fluid (CSF) analysis generally will be significant for albuminocytologic dissociation, or an elevated protein and normal to mildly elevated leukocyte count due to increased permeability BBB at proximal nerve roots [14]. An increased CSF protein concentration is highly dependent on the timing of the lumbar puncture after the onset of neurological symptoms, with one study involving 474 patients, finding protein elevation in only 49% on the first day to 88% after two weeks [14]. Despite this limitation, performing a lumbar puncture and CSF analysis is essential in assessing with wide differential associated with hyperacute GBS. Although not of significant importance in the ED setting, the diagnosis of AMAN is evidenced by confirmatory electrodiagnostic nerve conduction studies showing axonal involvement as presented by a reduction of compound muscle action potential amplitudes [4].

Respiratory monitoring is essential to the care of GBS in the ED. Patients presenting within the first week of symptoms should be monitored for the progression of their disease. As seen in our patient, neuromuscular weakness resulting in respiratory failure can occur rapidly requiring intubation, especially in rarer hyperacute presentations [6]. This necessitates close airway monitoring for signs of respiratory decompensation, such as worsening dyspnea, use of accessory respiratory muscles, bulbar dysfunction with swallowing impairment, inability to clear secretions, and signs of severe proximal muscle weakness, such as inability to lift the head or elbows [9]. Typical respiratory parameters that may indicate the need for pre-emptive intubation include significant tachypnea, Oxygen saturation of <92%, a forced vital capacity of <20 mL/kg or reduction >30% from prior measurement, and acute hypercapnia with a partial pressure of arterial carbon dioxide (PaCO2) >50 mmHg on blood gas analysis [9]. Providers should monitor for post-intubation hypotension due to the labile blood pressure induced by dysautonomia [9]. Of note, non-invasive bilevel-positive airway pressure may not be effective in GBS and may worsen dysautonomia [9,15]. Succinylcholine should be avoided given the well-known risk of inducing hyperkalemia.

Treatment of GBS involves IVIg or plasma exchange and supportive care for hemodynamic instability. plasma exchange and IVIg are the two primary treatments for adult and pediatric patients with GBS and are indicated for patients with severe cases of GBS, or those who are unable to walk without assistance [12]. In the ED, unless patients have a previous history of severe anaphylactic response to IVIg or anti-IgA antibodies and selective IgA deficiencies, IVIg with the typical standard regimen of 0.4 g/kg per day, for five consecutive days, and is typically preferred given the ease of administration [12]. As in our case, patients with an inadequate response after the initial dose may require a second course of IVIg [12]. Plasma exchange can also be utilized for patients in the acute phase with an inability to ambulate unless the patient cannot tolerate central line placement or with a history of allergy to frozen plasma [12]. Corticosteroid therapy and combined treatment of both plasma exchange and IVIg have shown no significant difference in patient outcomes compared to either therapy given alone; however, a method involving plasma exchange sessions alternating with IVIg infusions has shown potential in reducing mortality in pediatric patients [12]. In addition to primary treatment, supportive treatment for hemodynamic instability secondary to dysautonomia is essential. Dysautonomia, occurring in about 70% of cases, with about one-fifth with severe dysfunction, can include ileus, hypertension or hypotension, tachycardia or bradycardia as well as urinary retention [16]. These complications are treated supportively, with intravenous fluids and potentially vasopressors for hypotension, labetalol, nicardipine for hypertension, and neostigmine for ileus when without bradycardia [17-19]. Serious or life-threatening cardiac arrhythmias, including atrioventricular block and asystole, can occur with GBS and may require intervention atropine or cardiac pacing [17]. Dysautonomia correlates with a more severe peripheral nervous system involvement, including quadriparesis, bulbar, and neck flexor weakness, leading to greater morbidity and mortality [7-8,11,16].

## Conclusions

GBS is a rare ED presentation that can progress to life-threatening respiratory failure. Cases of GBS can be complicated by rarer variants with the potential to induce rapid, respiratory decompensation in the ED requiring emergent intubation. ED providers should understand the historic and exam findings associated with GBS, maintaining a high index of suspicion for rarer, rapidly progressive forms of GBS, and implement close respiratory monitoring for pre-emptive intubation.
